# Biomimetic Synthesis of Silver Nanoparticles Using Endosymbiotic Bacterium Inhabiting *Euphorbia hirta* L. and Their Bactericidal Potential

**DOI:** 10.1155/2016/9020239

**Published:** 2016-06-14

**Authors:** Baker Syed, Hoovinakola Chinnappa Yashavantha Rao, Mysore Nagalingaswamy Nagendra-Prasad, Ashwini Prasad, Ballagere Puttaraju Harini, Pasha Azmath, Devaraju Rakshith, Sreedharamurthy Satish

**Affiliations:** ^1^Bionanotechnological Laboratory, Department of Studies in Microbiology, University of Mysore, Manasagangotri, Mysore, Karnataka 570006, India; ^2^Department of Biotechnology, Sri Jayachamarajendra College of Engineering, JSS Technical Institutional Campus, Mysore 570006, India; ^3^Division of Microbiology, Faculty of Life Sciences, JSS University, Mysore, India; ^4^Department of Zoology, Bangalore University, Jnanabharathi Campus, Bangalore, Karnataka 560056, India

## Abstract

The present investigation aims to evaluate biomimetic synthesis of silver nanoparticles using endophytic bacterium EH 419 inhabiting* Euphorbia hirta* L. The synthesized nanoparticles were initially confirmed with change in color from the reaction mixture to brown indicating the synthesis of nanoparticles. Further confirmation was achieved with the characteristic absorption peak at 440 nm using UV-Visible spectroscopy. The synthesized silver nanoparticles were subjected to biophysical characterization using hyphenated techniques. The possible role of biomolecules in mediating the synthesis was depicted with FTIR analysis. Further crystalline nature of synthesized nanoparticles was confirmed using X-ray diffraction (XRD) with prominent diffraction peaks at 2*θ* which can be indexed to the (111), (200), (220), and (311) reflections of face centered cubic structure (*fcc*) of metallic silver. Transmission electron microscopy (TEM) revealed morphological characteristics of synthesized silver nanoparticles to be polydisperse in nature with size ranging from 10 to 60 nm and different morphological characteristics such as spherical, oval, hexagonal, and cubic shapes. Further silver nanoparticles exhibited bactericidal activity against panel of significant pathogenic bacteria among which* Pseudomonas aeruginosa* was most sensitive compared to other pathogens. To the best of our knowledge, present study forms first report of bacterial endophyte inhabiting* Euphorbia hirta* L. in mediating synthesizing silver nanoparticles.

## 1. Introduction

A substantial increase in microbial infection owing to rapid expansion of drug resistant microbial pathogens is rudimentary due to inadequate discoveries in the field of antimicrobial agents [1–3]. Scientific literatures have highlighted the severity of drug resistant pathogens which has created alarming situation across the globe leading to the need for novel antimicrobial agents. Hence, scientific communities are designing rational strategies in developing potent antimicrobial agents [4, 5]. In recent decades, protruding scientific interest illuminates new scientific domain nanotechnology which has demonstrated perpetual copious research in all fields of science that has influenced all forms of lives [6–8]. Interestingly, use of nanoparticles is reported to aid microbial infection by acting efficiently as antimicrobial agents [9]. Evaluation of nanoparticles as antimicrobial agents can form one of the potential alternative strategies towards combating drug resistant microorganisms especially silver nanoparticles. Role of silver is well documented since millennia and in recent years its applications have been rapidly expanded ever since the reports of silver and gold nanoparticles emerged [10–12]. Perusal of scientific literatures suggests that these nanoparticles act on pathogenic microorganisms with its multiple modes of actions; for instance nanoparticles are reported to interact with cell wall causing pits which results in loss of cellular contents, binding to thiol group of vital components, and damaging the DNA which in turn suppresses the replication process to name a few [13]. In a technical world these nanoparticles are playing significant role with myriad applications in multidisciplinary field of sciences [14]. Nanoparticles can be synthesized* via* various conventional methods but these methods are bound with various limitations such as generation of high heat, high cost, requiring high end instrumentation, and use of toxic elements in synthesis protocols creating serious concerns [15]. Hence in recent years, there is growing interest towards facile synthesis of nanoparticles which can be achieved by employing various biological entities which may vary from simple prokaryotic bacteria to eukaryotic fungi including plants [16, 17]. But one of the major constrains in employing plants results in harvesting of endangered species which can pose imbalance to plant diversity [18, 19]. Microorganisms form one of the inexhaustible and reliable sources which are reported to perform myriad biological functions [20]. One such important biological function includes remediation of toxic metals which can be traced down since ancient era [21]. This property of microorganisms has led to foundation stone towards synthesizing nanoparticles. Even though there has been extensive research on microbial mediated synthesis of nanoparticles, scanty reports are available on synthesis of nanoparticles from endophytes [8]. Endophytes are microorganisms which reside inside healthy tissue of almost all plant species and are reported to perform innumerable biological application and influences plant growth and development [22]. Endophytes secrete unique bioactive metabolites which are reported to have high significance and majority of the endophytes are yet to be explored [23]. Interference of endophytes with nanoparticles is one of the interesting areas which can open new avenues in reporting novel applications [24]. In the present investigation bacterial endophytes were screened from medicinal plant* Euphorbia hirta* L. and evaluated for synthesis of nanoparticles. Selection of plant species was carried out based on the previous report on endophytes and the medicinal properties of plants. Scientific studies demonstrate that* Euphorbia hirta* L. possesses medicinal properties that aid in treatment of gastrointestinal disorders and possess antioxidant, anti-inflammatory, and antimicrobial properties, anticancer activity, and nematicidal activity properties [25, 26]. Based on these considerations the present study was executed to isolate bacterial inhabiting* Euphorbia hirta* L. endophytes for synthesis of nanoparticles. To the best of our knowledge, this is the first preliminary report for synthesis of nanoparticles from bacterial endophyte isolated from* Euphorbia hirta* L.

## 2. Methodology

### 2.1. Sample Collection and Processing

*Euphorbia hirta* L. was collected from Srirangapatna is historical rocky island formed by Cauvery River with area 13 km^2^ (5 sq mi), situated 15–20 km from Mysore, Karnataka, India. Geographical coordinates are 12.41° North and 76.7° East. It has an average elevation of 679 m (2227 ft). The materials were collected in sterilized polythene bags and transported to laboratory. Collected plant materials were thoroughly washed under running tap water and then were immersed in double distilled water containing 50 *μ*g/mL of cycloheximide for 60 minutes [24].

### 2.2. Surface Sterilization Protocol

Plant materials were subjected to surface sterilization under aseptic condition and washed thoroughly with tap water followed by distilled water to remove adhering soil and debris. Later the surface sterilization was carried by sequential steps initially by immersing in 3.15% sodium hypochlorite for 120 seconds followed by 70% ethanol for 60 seconds and dried using sterile blotter sheets for 30 seconds. In every step of the surface sterilization procedures, the plant materials were washed in sterile double distilled water. To confirm that the surface disinfestations process was successful and to verify that there was no biological contamination from the surface sterilized plant segments, sterility checks were carried out for each sample to monitor the effectiveness by impressions and 0.1 mL from the final rinse was plated out on nutrient agar as control plate [27–29]. Colonies emerging from surface sterilized plant segments were subcultured and maintained with alphanumeric codes.

### 2.3. Screening of Endophytic Bacteria for Synthesis of Nanoparticles

Endophytic bacteria were cultured in the media incorporated with silver nitrate and incubated at 37°C until visible growth was observed. Further the colonies emerging from this enriched media were cultured in nutrient broth and incubated for 72 hours. The culture broth was centrifuged at 10,000 ×g at 4°C for 5 minutes and supernatant was assessed for synthesis of nanoparticles by challenging 1 mM of silver nitrate and incubated until color change was observed. Samples were drawn periodically and monitored using UV-Visible spectrophotometry to confirm the synthesis of nanoparticles [14].

### 2.4. Optimization Parameters for the Synthesis of Nanoparticles

The reaction mixture of the silver nitrate with cell-free supernatant of selected isolate was incubated at different temperatures ranging from 30°C to 80°C and the synthesis of nanoparticles was monitored by drawing the samples periodically and analyzing it using UV-Visible spectrophotometry. Effect of concentration of silver nitrate was optimized by varying concentration of silver nitrate ranging from 1.0 to 2.5 mM and ratio of supernatant with metal salts was studied to determine the optimal ratio required for rapid synthesis. The synthesis of nanoparticles was monitored by drawing the samples periodically and analyzing it using UV-Visible spectrophotometry. Effect of pH influencing the nanoparticles synthesis was carried out by varying the pH of the reaction mixture from 6 to 9. The synthesis of nanoparticles was monitored by drawing the samples periodically and analyzing it using UV-Visible spectrophotometry [14].

### 2.5. Biophysical Characterization of Nanoparticles

Samples were drawn and periodically monitored with UV-Visible spectroscopy by recording the spectra between 200 and 700 nm using Shimadzu double beam spectrophotometer. FTIR spectroscopy analysis conferred functional group of biomolecules responsible for mediating the synthesis on a JASCO FT-IR 4100 instrument at room temperature with a resolution of 4 cm^−1^. For XRD studies, nanoparticles were coated on XRD grid and Rigaku Miniflex-II Desktop X-ray diffractometer instrument operating at a voltage of 30 kV and average size was calculated based on Scherrer equation recorded spectra: *N* = *Kλ*/*β*cos⁡*θ*, where *K* is the Scherrer constant with value from 0.9 to 1 (shape factor), where *λ* is the X-ray wavelength (1.5418 Å), *β*1/2 is the width of the XRD peak at half height, and *θ* is the Bragg angle. Size and morphology of nanoparticles were analyzed by using Transmission Electron Microscopy; an aliquot of nanoparticles was transferred onto carbon-coated copper TEM grids. The films on the TEM grids were allowed to stand for 2 minutes and then extra solution was removed and the grid was allowed to dry prior to measurement and scanned using a TECHNAI-T12 JEOL JEM-2100 Transmission electron microscope operated at a voltage of 120 kV with Bioten objective lens. Subsequently, the particle size was ascertained using a Gatan ccd Camera [14].

### 2.6. Bactericidal Activity of Synthesized Nanoparticles

Antimicrobial activity of synthesized nanoparticles was evaluated against important human pathogens and phytopathogens via disc diffusion assay. In brief prewarmed MHA (Mueller-Hinton agar) plates were seeded with 10^6^ CFU (colony forming unit) suspensions of test organism which was swabbed uniformly and sterile disc was impregnated with 50 *μ*L of 10 mg/mL nanoparticles and incubated at 37°C for 24 hours. After incubation, the zone of inhibition was measured and interpreted with gentamicin [8]. All the test pathogens were procured from MTCC-IMTECH, Chandigarh, India.

## 3. Findings

**Figure 1:**
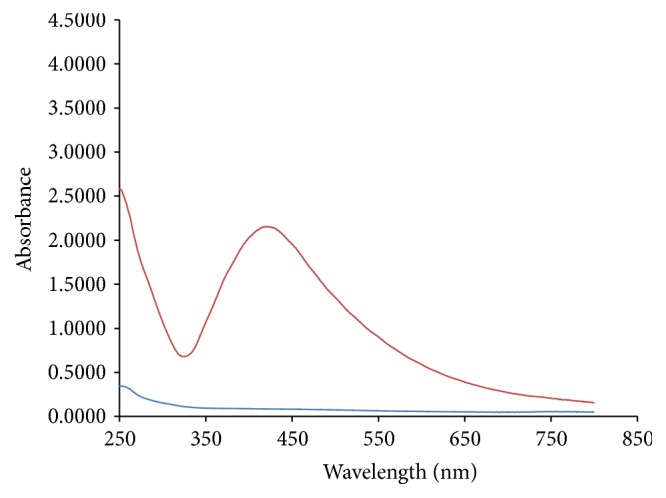
UV-Visible spectrum of synthesized silver nanoparticles by endophytic bacterium EH 419.

**Figure 2:**
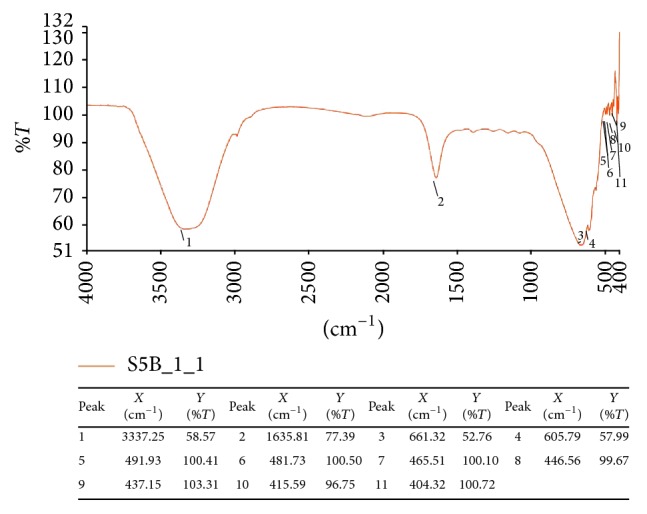
FTIR analysis of synthesized silver nanoparticles by endophytic bacterium EH 419.

**Figure 3:**
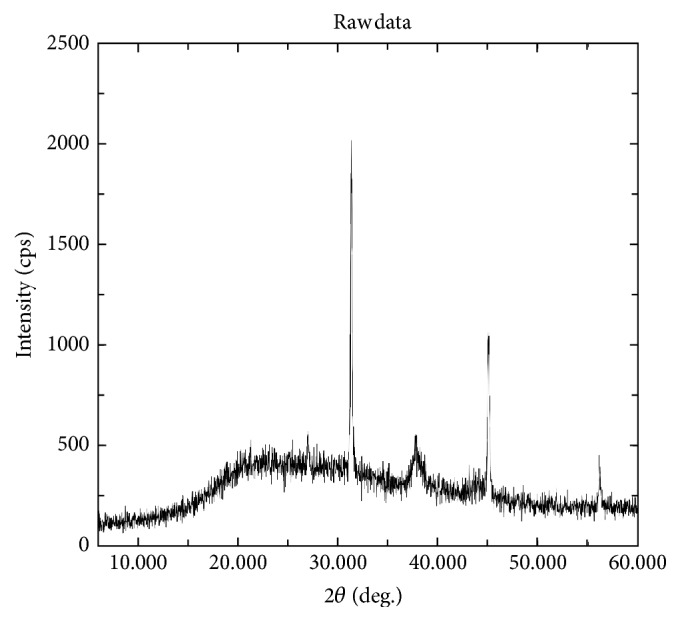
XRD analysis of synthesized silver nanoparticles by endophytic bacterium EH 419.

**Figure 4:**
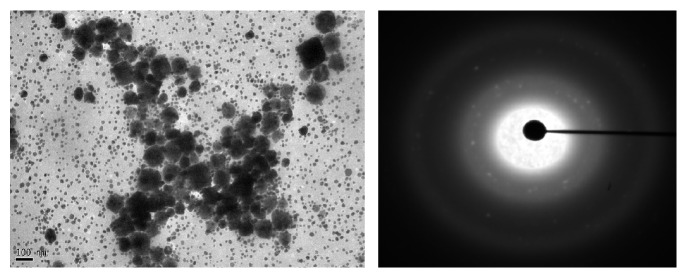
TEM analysis of synthesized silver nanoparticles by endophytic bacterium EH 419.

**Figure 5:**
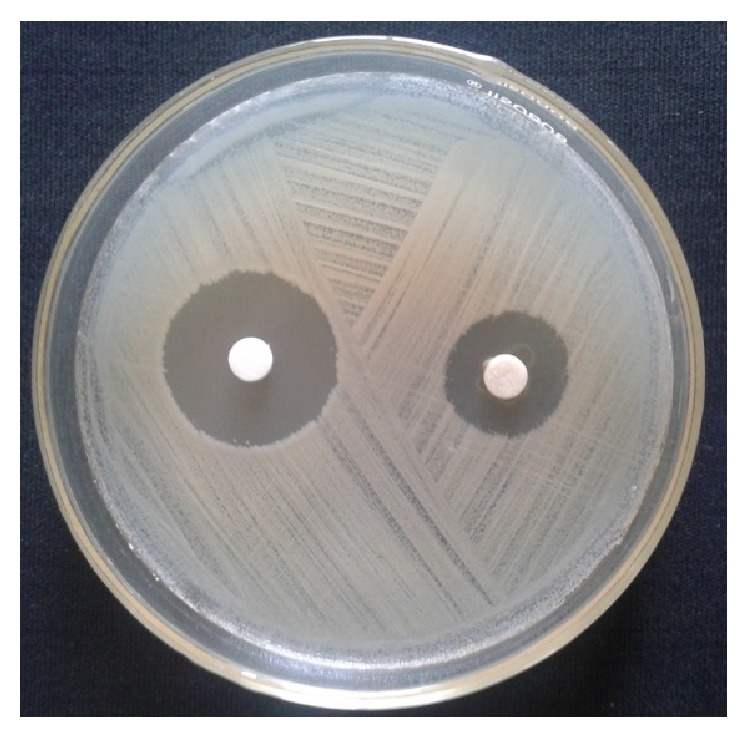
Bactericidal activity of synthesized silver nanoparticles by endophytic bacterium EH 419.

The results obtained in present investigation of use of surfactants like sodium hypochlorite and ethanol eliminated epiphytes from the plant materials. Further incorporation of cycloheximide resulted in suppression of fungal endophytes which resulted in only bacterial endophytes from plant segments. All the endophytic colonies were subcultured and evaluated for synthesis of nanoparticles by growing onto the media supplemented with metal salts. Majority of bacteria succumb to the toxicity of silver nitrate wherein only few bacteria are capable of growing. In the present investigation, only one bacterium was capable of growing luxuriantly which became the subject of interest in present investigation to carry out further experiments. The selected strain was assigned alphanumeric code EH 419 and was subjected to large-scale fermentation and cell-free supernatant was further assessed for synthesis of silver nanoparticles. Interestingly, the synthesis was rapid under optimized conditions under the influence of different parameters with elevated temperature above 70°C and alkaline pH influencing the synthesis of nanoparticles. The initial confirmation of synthesis was confirmed with gradual increase in the intensity of reaction mixture which resulted in dark brown color within 20 minutes of incubation time. Interestingly, no change in color was observed after 20 minutes indicating the attenuation of saturation point. Further confirmation of synthesis was achieved with UV-Visible spectrometry displaying prominent absorption peak conferring at 440 nm (Figure 1). This red shift in the absorption peaks is due to surface plasmon of synthesized silver nanoparticles. Further biophysical characterization of synthesized nanoparticles was carried out using hyphenated spectroscopic techniques. FTIR analysis (Figure 2) predicted the functional group responsible for mediating the synthesis and stabilization of nanoparticles. The broad absorbance band appearing at 3337 is due to OH group and the prominent peaks at 491 and 434 correspond to C-N and CH group. respectively. The XRD pattern of silver nanoparticles revealed the prominent diffraction peaks at 2*θ* which can be indexed to the (111), (200), (220), and (311) reflections of face centered cubic structure (*fcc*) of metallic silver (Figure 3). The average crystallite size “*d*” of silver nanoparticles was calculated to reveal the average size to be 30–35 nm using Scherer equation: *d* = *Kλ*/*β*cos⁡*θ*, where *K* is shape factor between 0.9 and 1.1, *k* is incident X-ray wavelength (Cu*Kα* = 1.542 Å), *β* is full width half-maximum in radians of the prominent line, and *θ* is position of that line in the pattern. The TEM micrographs of precipitated solid phase revealed the size ranging from 10 to 60 nm with an average size of 30 nm and shapes of the silver nanoparticles (Figure 4). Synthesized silver nanoparticles were polydispersed in nature with different morphological characteristics such as spherical, oval, hexagonal, cubic, and triangular shapes. Biologically synthesized silver nanoparticles exhibited bactericidal activity against panel of selected pathogens via disc and well diffusion assay. The bactericidal activity was determined by measuring zone of inhibition across the well and disc (Table 1 and Figure 5) and highest activity was conferred against* Pseudomonas aeruginosa* (MTCC 7903) followed by* Escherichia coli* (MTCC 7410),* Staphylococcus aureus* (MTCC 7443),* Bacillus subtilis* (MTCC 121), and* Klebsiella pneumoniae* (MTCC 7407).

## 4. Discussion

Endophytes are of the highly merited microbial sources owing to its adaptation to unique biological niches in higher plants. Studies also confer that these endophytes are capable of secreting structurally diverse classes of secondary metabolites bearing activities [30]. In present investigation, bacterial endophytes inhabiting* Euphorbia hirta* L. were screened for synthesis of silver nanoparticles. Surface sterilization process was successful with no growth on control plate and supplementation of antifungal agent suppressed growth of fungal endophytes. The sterilization protocol was conducted based on the earlier protocols and standardization. In the present investigation, preliminary screening of endophytes for synthesis resulted in selection of endophytic bacterium EH 419 based on luxuriant growth on metal incorporated media. To the best of our knowledge, this is the first preliminary report for synthesis of nanoparticles from endophyte isolated from* Euphorbia hirta* L. Even though there are large scientific studies pertaining to microbial synthesis of nanoparticles, scanty reports are available on bacterial endosymbionts capable of synthesizing nanoparticles; hence in the present investigation bacterial endophytes became the subjected of interest. The selected endophytic bacterium was subjected to large-scale fermentation to obtained cell-free extract via centrifugation to assess extracellular synthesis of silver nanoparticles. The process of extracellular synthesis is advantageous compared to intracellular synthesis, which results in one-step synthesis protocol [8]. During the synthesis of nanoparticles, it was observed that elevated temperature and alkaline pH influenced the synthesis. This result is in agreement with earlier findings, which highlight the importance of parameters [31]. The change in color from pale yellow to dark brown color is attributed to surface plasmon resonance which causes red shift as observed in Figure 1; these results justify previous findings [32]. Generally, the stability of silver nanoparticles is more significant for its applicative point of view especially in biomedicine. Consequently, the silver nanoparticles are normally stabilized by using some stabilizing agents. However, in present investigation, synthesized silver nanoparticles were more stable owing to* in situ* biocapping which is observed with FTIR analysis (Figure 2) with various functional groups responsible for mediating and stabilizing nanoparticles. Interestingly these results also coincide with the majority of earlier findings, which state that presence of amides, aliphatic, carbonyl, and aromatic groups mediated the synthesis and stabilization of nanoparticles [8, 33]. The XRD patterns revealed the crystalline nature of synthesized silver nanoparticles based on the diffraction pattern, which coincides with the previous scientific reports [32, 34, 35]. The morphological characteristics of synthesized nanoparticles exhibited polydispersity of nanoparticles with myriad shapes. These characteristics of silver nanoparticles are highly essential for fate of their applications and obtained results are in agreement with previous biosynthesized nanoparticles [36]. The synthesized silver nanoparticles exhibited significant bactericidal activity against targeted pathogens. Use of silver as potent antimicrobial agents is well documented with silver based products and with the invention of silver nanoparticles; the activity has been more profound especially against multidrug resistant microorganisms. The bactericidal activity of synthesized silver nanoparticles was assessed via disc diffusion, well diffusion, and broth dilution assay which resulted in* Pseudomonas aeruginosa* (MTCC 7903) being more sensitive compared to other test pathogens. Scientific report suggests that pathogenic* Pseudomonas aeruginosa* is one of the deleterious microbial pathogens infecting all forms of lives thus serving as human pathogens and phytopathogens. These results justify the earlier findings which report evaluation of silver nanoparticles as potent antimicrobial agents [37–39]. Overall, The results obtained in present investigation are promising enough and attribute towards the growing knowledge on endophytes and their untraced roles [40].

## 5. Conclusion

The present study reports biomimetic synthesis of silver nanoparticles using endosymbiotic bacterium inhabiting* Euphorbia hirta* L. and their bactericidal potential. Scanty reports are available on evaluation of endophytes for synthesis of nanoparticles; present study forms first report on synthesis of nanoparticles from endophytic bacterium EH419 isolated from* Euphoria hirta* L. The obtained results are promising enough to report preliminary investigation and future molecular characterization is highly essential to reveal the affiliation of selected endophyte.

## Supplementary Material

The supplementary material highlights the overall schematic representation of endophytic bacteria in synthesis of silver nanoparticles.

## Figures and Tables

**Table 1:** Bactericidal activity of silver nanoparticles via disc diffusion assay.

Serial number	Test pathogens	Silver nanoparticles	Gentamicin (1 mg/mL)
1	*Bacillus subtilis *(MTCC 121)	13.00 mm	34.00 mm
2	*Escherichia coli *(MTCC 7410)	15.00 mm	29.00 mm
3	*Klebsiella pneumoniae *(MTCC 7407)	11.00 mm	18.00 mm
4	*Pseudomonas aeruginosa *(MTCC 7903)	20.00 mm	24.00 mm
5	*Staphylococcus aureus *(MTCC 7443)	14.00 mm	24.00 mm

## References

[1] Lin Y.-T., Wang Y.-P., Wang F.-D., Fung C.-P. (2015). Community-onset *Klebsiella pneumoniae* pneumonia in Taiwan: clinical features of the disease and associated microbiological characteristics of isolates from pneumonia and nasopharynx. *Frontiers in Microbiology*.

[2] Khan H. A., Ahmad A., Mehboob R. (2015). Nosocomial infections and their control strategies. *Asian Pacific Journal of Tropical Biomedicine*.

[3] Nurain A. M., Bilal N. E., Ibrahim M. E. (2015). The frequency and antimicrobial resistance patterns of nosocomial pathogens recovered from cancer patients and hospital environments. *Asian Pacific Journal of Tropical Biomedicine*.

[4] Ma Q. Q., Lv Y. F., Gu Y., Dong N., Li D. S., Shan A. S. (2013). Rational design of cationic antimicrobial peptides by the tandem of leucine-rich repeat. *Amino Acids*.

[5] Pathak S., Chauhan V. S. (2011). Rationale-based, de novo design of dehydrophenylalanine-containing antibiotic peptides and systematic modification in sequence for enhanced potency. *Antimicrobial Agents and Chemotherapy*.

[6] Baker S., Satish S. (2015). Biosynthesis of gold nanoparticles by *Pseudomonas veronii* AS41G inhabiting *Annona squamosa* L.. *Spectrochimica Acta A: Molecular and Biomolecular Spectroscopy*.

[7] Makarov V. V., Love A. J., Sinitsyna O. V. (2014). Green nanotechnologies: Synthesis of metal nanoparticles using plants. *Acta Naturae*.

[8] Azmath P., Baker S., Rakshith D., Satish S. (2016). Mycosynthesis of silver nanoparticles bearing antibacterial activity. *Saudi Pharmaceutical Journal*.

[9] Ravishankar Rai V., Bai A. J., Méndez-Vilas A. (2011). Nanoparticles and their potential application as antimicrobials. *Science against Microbial Pathogen: Communicating Current Research and Technological Advances*.

[10] Arvizo R. R., Bhattacharyya S., Kudgus R. A., Giri K., Bhattacharya R., Mukherjee P. (2012). Intrinsic therapeutic applications of noble metal nanoparticles: past, present and future. *Chemical Society Reviews*.

[11] Franci G., Falanga A., Galdiero S. (2015). Silver nanoparticles as potential antibacterial agents. *Molecules*.

[12] Matthews L., Kanwar R. K., Zhou S., Punj V., Kanwar J. R. (2010). Applications of nanomedicine in antibacterial medical therapeutics and diagnostics. *Open Tropical Medicine Journal*.

[13] Buzea C., Pacheco I. I., Robbie K. (2007). Nanomaterials and nanoparticles: sources and toxicity. *Biointerphases*.

[14] Baker S., Kumar K. M., Santosh P., Rakshith D., Satish S. (2015). Extracellular synthesis of silver nanoparticles by novel *Pseudomonas veronii* AS41G inhabiting *Annona squamosa* L. and their bactericidal activity. *Spectrochimica Acta Part A: Molecular and Biomolecular Spectroscopy*.

[15] Baker S., Harini B. P., Rakshith D., Satish S. (2013). Marine microbes: invisible nanofactories. *Journal of Pharmacy Research*.

[16] Baker S., Rakshith D., Kavitha K. S. (2013). Plants: emerging as nanofactories towards facile route in synthesis of nanoparticles. *BioImpacts*.

[17] Iravani S., Korbekandi H., Mirmohammadi S. V., Zolfaghari B. (2014). Synthesis of silver nanoparticles: chemical, physical and biological methods. *Research in Pharmaceutical Sciences*.

[18] Baker S., Kavitha K. S., Rao H. C. Y. (2015). Bacterial endo-symbiont inhabiting *Tridax procumbens* L. and their antimicrobial potential. *Chinese Journal of Biology*.

[19] Baker S., Satish S. (2012). Endophytes: toward a vision in synthesis of nanoparticle for future therapeutic agents. *International Journal of Bio-Inorganic Hybrid Nanomaterials*.

[20] Staniek A., Woerdenbag H. J., Kayser O. (2008). Endophytes: exploiting biodiversity for the improvement of natural product-based drug discovery. *Journal of Plant Interactions*.

[21] Gunasekaran P., Muthukrishnan J., Rajendran P. (2003). Microbes in heavy metal remediation. *Indian Journal of Experimental Biology*.

[22] Strobel G. A. (2003). Endophytes as sources of bioactive products. *Microbes and Infection*.

[23] Alvin A., Miller K. I., Neilan B. A. (2014). Exploring the potential of endophytes from medicinal plants as sources of antimycobacterial compounds. *Microbiological Research*.

[24] Baker S., Sahana S., Rakshith D., Kavitha K. U., Kavitha K. S., Satish S. (2012). Biodecaffeination by endophytic *Pseudomonas* sp. isolated from *Coffee arabica* L. *Journal of Pharmacy Research*.

[25] Upadhyay B., Singh K. P., Kumar A. (2010). Pharmacognostical and antibacterial studies of different extracts of *Euphorbia hirta* L.. *Journal of Phytopathology*.

[26] Huang L., Chen S., Yang M. (2012). Euphorbia hirta (Feiyangcao): a review on its ethnopharmacology, phytochemistry and pharmacology. *Journal of Medicinal Plants Research*.

[27] Webster N. S., Wilson K. J., Blackall L. L., Hill R. T. (2001). Phylogenetic diversity of bacteria associated with the marine sponge rhopaloeides odorabile. *Applied and Environmental Microbiology*.

[28] Zin N. M., Loi C. S., Sarmin N. M., Rosli A. N. (2010). Cultivation-dependent characterization of endophytic actinomycetes. *Research Journal of Microbiology*.

[29] Baker S., Satish S. (2012). Antimicrobial activity and biosynthesis of nanoparticles by endophytic bacterium inhabiting *Coffee arabica* L.. *Scientific Journal of Biological Sciences*.

[30] Yashavantha Rao H. C., Santosh P., Rakshith D., Satish S. (2015). Molecular characterization of an endophytic *Phomopsisliquidambaris* CBR-15 from *Cryptolepis buchanani* Roem. and impact of culture media on biosynthesis of antimicrobial metabolites. *3 Biotech*.

[31] Qian Y., Yu H., He D. (2013). Biosynthesis of silver nanoparticles by the endophytic fungus Epicoccum nigrum and their activity against pathogenic fungi. *Bioprocess and Biosystems Engineering*.

[32] Awwad A. M., Salem N. M., Abdeen A. O. (2013). Green synthesis of silver nanoparticles using carob leaf extract and its antibacterial activity. *International Journal of Industrial Chemistry*.

[33] Yilmaz M., Turkdemir H., Kilic M. A. (2011). Biosynthesis of silver nanoparticles using leaves of *Stevia rebaudiana*. *Materials Chemistry and Physics*.

[34] Ahmad A., Mukherjee P., Senapati S. (2003). Extracellular biosynthesis of silver nanoparticles using the fungus *Fusarium oxysporum*. *Colloids and Surfaces B: Biointerfaces*.

[35] Kathiresan K., Manivannan S., Nabeel M. A., Dhivya B. (2009). Studies on silver nanoparticles synthesized by a marine fungus, *Penicillium fellutanum* isolated from coastal mangrove sediment. *Colloids and Surfaces B: Biointerfaces*.

[36] Devi N. N., Shankar P. D., Sutha S. (2012). Biomimetic synthesis of silver nanoparticles from an endophytic fungus and their antimicrobial efficacy. *International Journal of Journal Biomedical Advance Research*.

[37] Sondi I., Salopek-Sondi B. (2004). Silver nanoparticles as antimicrobial agent: a case study on *E. coli* as a model for Gram-negative bacteria. *Journal of Colloid and Interface Science*.

[38] Fayaz A. M., Balaji K., Girilal M., Yadav R., Kalaichelvan P. T., Venketesan R. (2010). Biogenic synthesis of silver nanoparticles and their synergistic effect with antibiotics: a study against gram-positive and gram-negative bacteria. *Nanomedicine: Nanotechnology, Biology, and Medicine*.

[39] Otari S. V., Patil R. M., Ghosh S. J., Thorat N. D., Pawar S. H. (2015). Intracellular synthesis of silver nanoparticle by actinobacteria and its antimicrobial activity. *Spectrochimica Acta Part A: Molecular and Biomolecular Spectroscopy*.

[40] Sunkar S., Nachiyar C. V. (2012). Biogenesis of antibacterial silver nanoparticles using the endophytic bacterium *Bacillus cereus* isolated from *Garcinia xanthochymus*. *Asian Pacific Journal of Tropical Biomedicine*.

