# Supplementary figures and images for: Biomimetic Synthesis of Silver Nanoparticles Using Endosymbiotic Bacterium Inhabiting Euphorbia hirta L. and Their Bactericidal Potential
Source: Scientifica (Cairo). 2016 Jun 14;2016:9020239. doi: 10.1155/2016/9020239 (PMC4923596; doi:10.1155/2016/9020239)

## Graphical Abstract

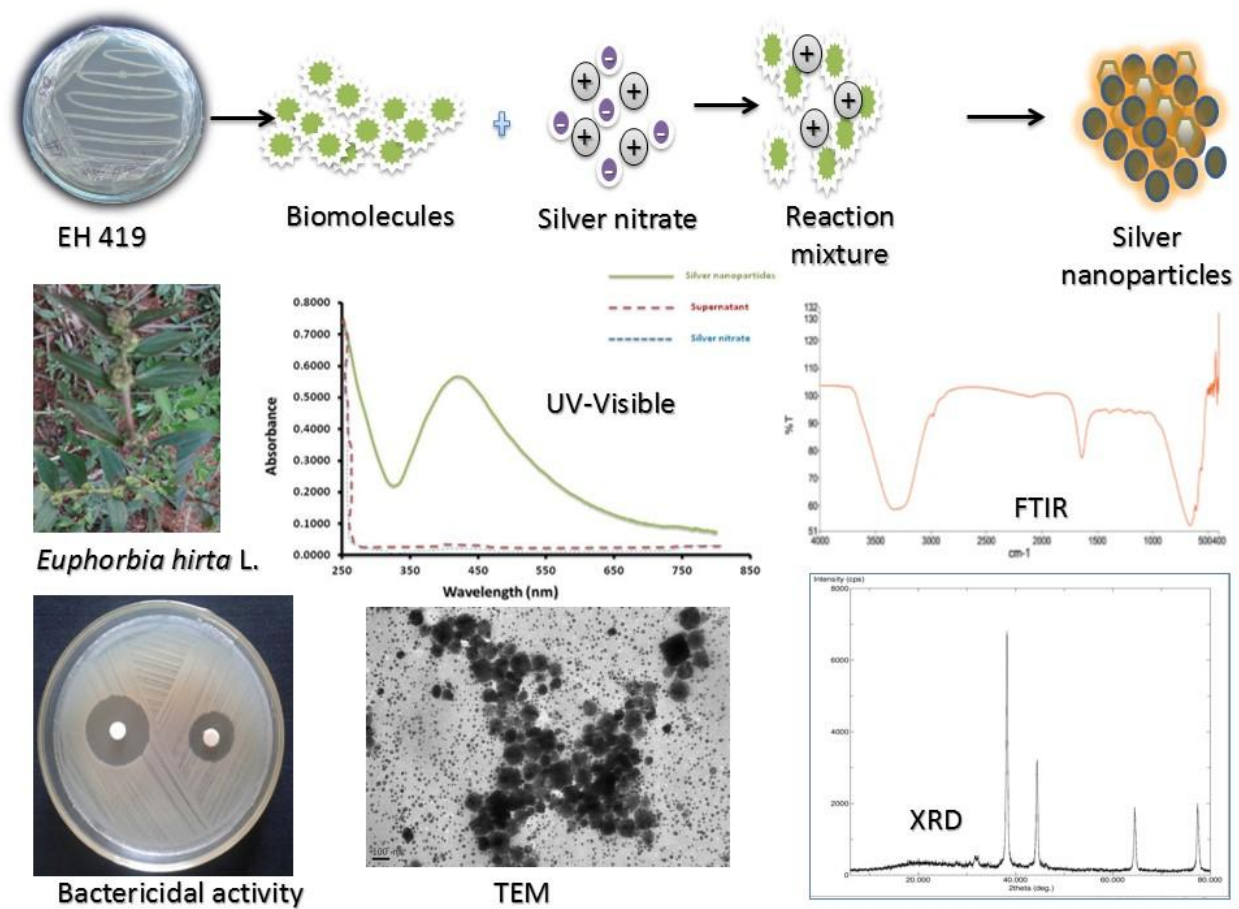

Supplement: Supplementary file 1 — The supplementary material highlights the overall schematic representation of endophytic bacteria in synthesis of silver nanoparticles. [file 9020239.f1.pdf]
